# Passionate hearts, torn loyalties: navigating the interplay between fandom and romance

**DOI:** 10.3389/fpsyg.2023.1240271

**Published:** 2023-10-10

**Authors:** Orr Levental, Dalit Lev Arey, Assaf Lev

**Affiliations:** ^1^Department of Physical Education, The Research Center for Sports and Physical Activity, Tel-Hai College, Tel Hai, Israel; ^2^Department of Psychology, Academic College of Tel Aviv-Yaffo, Tel Aviv-Yafo, Israel; ^3^Department of Sports Therapy, Faculty of Health Professions, Ono Academic College, Kiryat Ono, Tel Aviv, Israel

**Keywords:** sports fandom, Israel, romantic relationship, ardent fans, spouses

## Abstract

**Introduction:**

The current study presents a thorough investigation of the attitudes and emotions expressed by the spouses and girlfriends of sports fans within the context of their long-term relationships. Its primary objective is to explore the potential ramifications that surface when individuals become deeply invested in a competitive sport, demonstrating intense emotional connections, broad knowledge, unwavering loyalty, and active engagement in fan-related activities.

**Methods:**

The research methodology applied in this study involved conducting semi-structured interviews with a sample of twelve spouses and girlfriends of fans.

**Results:**

The principal findings elucidate four distinct themes that shed light on the intersection of fandom and romantic relationships. These themes include: “that’s what I signed up for”; “fandom as a gendered activity”; “the good, the bad, and the ugly”; and “sports fandom as an addiction.”

**Discussion:**

The study concludes by highlighting the crucial cultural context at work. The social criticism expressed by the interviewees toward non-traditional gender behaviors displayed by their partners serves as a cautionary message. These criticisms aim to preserve and uphold male dominance in sports, thereby reinforcing the notion of their unquestioned supremacy. Consequently, we argue that, within this context, latent aggressive behavior in men is perceived as a strategy to maintain their monopolistic control over sports domains.

## Introduction

A man can change anything: his face, his family, his girlfriend, his religion, his God. But there is something a man can not change. A man can not change his passion (Campanella, 2009).

This quote from the Argentinian film the secret in their eyes vividly captures the essence of an ardent sports fan’s commitment to their club. It portrays a lifestyle deeply infused with fandom, where their devotion is not merely a hobby, but also pervades their attitudes behaviors, and even their schedules Their fandom extends into their choice of friends, the colors they prefer for their clothing and other items, and their patterns of consumption. As such, the emotional, financial, and time investments that fans pour into their beloved club often come into conflict with other aspects of their daily lives. One such potential conflict, and the focus of this paper, is the impact of ardent fandom on romantic relationships. We explore how a fan’s significant other navigates the symbolic competition between themselves and the club. Accordingly, this paper aims to offer an initial exploration of the attitudes and emotions of fans’ spouses and girlfriends as they maintain long-lasting relationships with these passionate sports enthusiasts.

### Sports fans

Sports fandom is a widespread and enthralling phenomenon with considerable impact on contemporary society, engaging millions of individuals globally and inspiring a degree of commitment likened to religious devotion (Dwyer and Drayer, 2010; Tamir, 2018). To thoroughly understand sports fandom, it is crucial to consider its cognitive, affective, and behavioral dimensions. James and Ridinger (2002) proposed a significant framework defining a sports fan, emphasizing self-identification, supportive attitudes, and observable behaviors as key elements. Fans forge deep emotional bonds with the teams or individuals they support, nurturing a strong sense of unity. They actively indulge in consuming news, sharing experiences via social networks, and distinguishing themselves from those possessing only casual interest in a team or player (Hull and Lewis, 2014).

Various forms of fandom have been identified and categorized by researchers, predicated on the degree of identification fans exhibit toward their team (Giulianotti, 2002; Billings et al., 2018; Onwumechili, 2018; Van Driel et al., 2019; Levental et al., 2022). Wann et al. (2001) suggest a framework that differentiates highly identified fans from mere sports spectators. Highly identified fans are deeply committed to a specific sport, team, or athlete, demonstrating strong emotional attachment, extensive knowledge, steadfast loyalty, and active participation in fan activities (Levental et al., 2021b). In contrast, sports spectators display a more passive, casual interest, viewing sports events without the same intensity of emotional connection or active involvement. The team holds remarkable stability in the fans’ lives, often assuming priority in disputes with other identities (Porat, 2010).

### Characteristics of sports fans

The characteristics of sports fans have been extensively explored by researchers such as Wann et al. (2001) and James and Ridinger (2002) within the frameworks of sports fandom and fan identification. These frameworks are built upon psychological theories such as social identity theory (Tajfel and Turner, 1986) and self-determination theory (Deci and Ryan, 1985), enabling a comprehensive understanding of the motivations and behaviors exhibited by sports fans. The key features of a sports fan revolve around their motivations, identification, and behaviors (Wann et al., 2001; James and Ridinger, 2002).

Motivations for sports fandom encompass diverse factors, including seeking entertainment, experiencing eustress, forming group affiliations, enhancing self-esteem, appreciating esthetics, seeking escapism, valuing family connections, and considering economic factors (Funk et al., 2012). Men often find motivation in the excitement, arousal, self-worth, and aesthetic appeal of sports, while women are more motivated by social interactions and the opportunity to spend time with friends and family (Gantz and Wenner, 1991; Wann et al., 1999a,b, 2001). Additionally, research has identified other motivations such as empathy, seeking action, acquiring knowledge, and engaging in social interaction (Mastromartino and Zhang, 2020).

Identification plays a crucial role in sports fandom, indicating the psychological connection fans have with a specific team or sport. Highly identified fans demonstrate a strong sense of loyalty, knowledge, and attachment to fellow fans, often expressing their support through visible behaviors like wearing team colors (Fillis and Mackay, 2014). This identification with a team or sport becomes an integral part of a fan’s identity, fostering a sense of belonging and community within the fan base. It is important to recognize that identification extends not only to the team or sport but also to other fans, as fanship and fandom encompass both individual and collective aspects (Wann et al., 2001).

Fans’ behaviors in sports fandom encompass various activities such as attending games, cheering, acquiring knowledge, and displaying team colors (Wakefield and Sloan, 1995). Attendance at games is often associated with higher levels of loyalty. These behaviors allow fans to actively engage with their favorite teams or sports, showing their support and contributing to the overall enjoyment of being a sports fan (Wann et al., 2001; James and Ridinger, 2002).

### Hobbies and addiction

Engaging in sports fandom as a hobby can bring joy, entertainment, and a sense of fulfillment and meaning to individuals (Zhigang et al., 2022). It offers opportunities for social interaction and personal growth, as fans admire the skills and teamwork of athletes (Kim and Kim, 2020). However, it is essential to be aware of the potential for addictive behavior. Research has shown that activities like shopping, video gaming, television watching, internet surfing, texting, and even eating chocolate can transition from enjoyable pastimes to compulsions and addictions (Kubey and Csikszentmihalyi, 2004; Ridgway et al., 2008; Huang and Leung, 2009).

Compulsive sport consumption is characterized by habitual and obsessive engagement with sports, often with a sense of limited control (Aiken et al., 2018). It can lead to negative internal feelings such as guilt, regret, and conflict, as well as external consequences like strained relationships and professional life (Simmons et al., 2011). Compulsive sport consumption shares similarities with addiction, involving obsessive-compulsive tendencies and difficulties with impulse control (Huang and Leung, 2009). Several forces contribute to the development of excessive sport consumption as a compulsion. Internally, fan values, attitudes, and identities shape loyalty and positive self-esteem (Bee and Kahle, 2006; Aiken et al., 2018). Externally, social forces promote social cohesion and a sense of belonging, encouraging excessive sport consumption (Branscombe and Wann, 1991; Campbell et al., 2004). Media and marketing cues also play a role, bombarding fans through various channels and fostering engagement with diverse sports (Fullerton and Punj, 2004; Prentice and Cotte, 2015; Hing et al., 2016).

In today’s technology-driven society, where sports are easily accessible through multiple media platforms, many fans struggle to control their consumption and may prioritize it over other responsibilities (Gubar, 2015). Recognizing warning signs of excessive involvement in sports fandom, such as neglecting responsibilities, seeking constant validation from sports outcomes, or experiencing distress when unable to follow events, is crucial as they may indicate an unhealthy attachment (Mastromartino and Zhang, 2020).

### The conflict between fandom and family

Balancing a passion for sports with family commitments presents challenges for sports fans (Madsen and Hammond, 2006). Conflicts may arise when sports events clash with significant family occasions or when the time and emotional investment in sports compromise quality time with loved ones. Individuals have multiple roles, including being a parent, spouse, student, and sports fan, each demanding time, energy, and limited resources (Grawitch et al., 2010). These roles can conflict with each other, leading to interrole conflict (Greenhaus and Beutell, 1985). While sports fandom may initially seem harmless, it can become addictive and disrupt one’s life (Smith, 1988).

Dysfunctional fandom occurs when a fan’s being hinders fulfilling normal role behaviors outside of sports, negatively impacting family life and relationships (Simmons and Greenwell, 2014; Smith, 2017). Devoting significant time, money, and energy to the fan role may result in neglecting family obligations (Lapierre et al., 2008; Vallerand et al., 2008a). Conflicts between the fan and family roles can arise when sports events take precedence over family events or when negative emotions following a team loss affect interactions with loved ones (Wann et al., 2001; Wann and Waddill, 2013). Moreover, highly identified fans, deeply invested in their fan role, often spend more time and engage in support behaviors that contribute to interrole conflict (Trail et al., 2003; Wann and Waddill, 2013). The financial commitment associated with being a fan can create conflict within the family, as the resources allocated to the fan role may be seen as hindering financial obligations (Wann and Branscombe, 1993; Downs and Woolrych, 2010). Interrole conflict has a negative impact on family satisfaction and overall life satisfaction (Carlson and Kacmar, 2000; Judge et al., 2006).

Fan-family conflict arises when the time and emotional investment in being a sports fan clash with family commitments and relationships (Wenner, 2002). This conflict can manifest as neglecting family obligations or experiencing negative emotions that affect interactions with loved ones (Simmons and Greenwell, 2014), resulting in diminished relationship satisfaction and strained family functioning (John, 2004). Various types of fan-family conflict have been identified in the literature, including time-based, strain-based, behavior-based, and economic-based conflicts (Vallerand et al., 2008b; Laß and Wooden, 2023). Time-based conflict occurs when multiple roles compete for limited time, while strain-based conflict arises when stress from one role spills over and affects performance in another. Behavior-based conflict occurs when behavioral expectations of different roles are incompatible, and economic-based conflict arises when financial demands of the fan and family roles conflict (Downs and Woolrych, 2010).

### Sports fandom and gender in Israel

Considering the global nature of sports, sports fandom exhibits similar characteristics across different countries and among individuals. However, the dynamics of this relationship can be significantly influenced by local cultural and social contexts. In the case of Israel, where research on the impact of sports fandom on family life is limited, it is crucial to examine this phenomenon within the unique gender perceptions prevalent in society. Scholarly investigations into the relationship between sports and gender in Israel primarily focus on examining gender power dynamics and their influence on identity formation. Kay and Jeanes (2010) argue that as women increasingly participate in traditionally male-dominated professions and activities, sports have become an important arena for gender-related struggles.

The discourse surrounding women’s involvement in sports in Israel, like in other spheres, often marginalizes their activities compared to those of men. Discriminatory language emerges in discussions that differentiate and prioritize various activity categories, using terms such as “politics” versus “women’s politics,” “empowerment” versus “women’s empowerment,” and “leadership” versus “women’s leadership.” This gender bias is pervasive in the realm of sports and reinforces the intertwining of masculinity with national identity. As a result, male-dominated sports continue to be regarded as the unquestioned standard of excellence (Lev and Hertzog, 2022). Furthermore, it is important to highlight that male engagement in sports activities often adopts military symbolism, serving as a formative and empowering metaphor (Lev and Hertzog, 2017; Hertzog and Lev, 2019). This association is not surprising given the significant role of military service in the maturation process of young Israelis, which shapes their consciousness and blurs the boundaries between the individual, family, society, nation, and state (Ben-Eliezer, 1995; Sasson-Levy and Hartal, 2018).

In Israel, sports fandom also holds a prominent and integral place within the cultural and social tapestry (Levental et al., 2021a; Tamir and Galily, 2021), reflecting a robust interest in various sports, including football, basketball, and martial arts, underscoring its profound role in society (Galily and Samuel-Azran, 2021). This fervor is ignited by the accomplishments of national teams and the prominence of local leagues (Porat, 2010; Lev and Weinish, 2020). Sports teams serve as unifying symbols within Israel’s diverse and multicultural community, adding depth and complexity to the experiences of sports enthusiasts (Lev and Zach, 2018). This unique Israeli context not only showcases the passion for sports but also provides a rich foundation for scrutinizing the dynamics of sports fandom and its far-reaching societal implications. In essence, it exemplifies how sports, like football on a global scale, transcends boundaries and serves as a powerful force that connects people from various backgrounds and walks of life.

## Materials and methods

### Instrumentation and participants

To explore how spouses and girlfriends of avid sports fans perceive their relationships, we conducted semi-structured interviews with twelve individuals involved with ardent sports enthusiasts. We define ardent fans as sports supporters who spend, on average, at least 2 h a day engaged in fan-related activities, such as attending games and practices or participating in fan committees. We adopted Giulianotti’s (2002) characterization of ardent sports supporters as fans who demonstrate prolonged support and solidarity for a team, displaying unconditional love for their team and investing considerable time and emotions throughout the year.

The first two interviewees were approached based on personal acquaintance of the researchers. After the interview, the snowball method was used to locate additional interviewees. It should be noted that this sampling method is particularly effective when the research topic is related to personal aspects or related to a closed social group (Noy, 2008). The participants in this study were Israeli women aged between 26 and 40, each in a relationship for at least a year with a male ardent sports fan. These women were asked primarily about their perceptions, beliefs, and expectations regarding their relationships. Sample questions included, “How does it feel to be in a relationship with an ardent sports fan?” and “To what degree are you involved in your spouse’s sports world?”

Each interview ranged from 60 to 90 min in duration and was conducted by the researchers. Interviews took place in various locations, either in a quiet coffee shop or at the interviewees’ homes. They were all recorded and initially conducted in Hebrew but were subsequently translated into English by the researchers for analysis. From these interviews, four overarching themes emerged: “that’s what I signed up for”; “fandom as a gendered activity”; “the good, the bad, and the ugly”; and “sports fandom as an addiction.” The study concludes by underlining the significant influence of cultural context in shaping these dynamics.

### Data analysis

To analyze the data, we employed the thematic analysis method (Braun et al., 2016). This approach allows for the identification, interpretation, and explanation of themes within a dataset. The researchers conducted an immersive exploration of the data by diving into the transcripts, coding the data, and generating themes. We adopted an inductive approach where themes and patterns were allowed to emerge organically from the data itself. Moreover, we engaged with the data at the level of semantic focus. Through the organization of data into higher-order themes, certain codes were examined and subsequently amalgamated to shape overarching themes. The overarching themes identified were: sports fandom perpetuating gender inequality within the family; the “love and hate” relationship inspired by sports fandom; and the implications of ardent sports fandom within the relationship. Following the creation of these themes, we aimed to establish coherent connections among the categories. This required an exploration of the relationships between the themes and an understanding of how they interlinked to form a broader representation of the data.

As we described and reflected on these themes, we utilized existing literature to link them to the broader human experience of ardent sports fandom within the socio-cultural perspective, thereby providing a comprehensive view of the findings.

### Assurance of quality

Ethical considerations necessitated several steps, as recommended by scholars such as Birt et al. (2016). These steps included obtaining signed consent forms from the participants, indicating their voluntary agreement to participate in the study, as well as conducting member checking to seek the participants’ validation of the data analysis. Member checking was employed to enhance the credibility and trustworthiness of the research findings. Although this process can be viewed as intricate and potentially controversial, careful attention was given to providing participants with an open platform for commenting and criticizing. The first author of the study facilitated this process by sharing audio recordings and draft accounts of the interviews with the participants, and their input was incorporated into the final published paper. By employing this approach, participants were afforded the opportunity to review and approve their contributions throughout the research process (Creswell, 2009). However, it is important to note, following the guidance of Sparkes and Smith (2013), that the author did not consider participants’ feedback as direct validation or refutation of their inferences. Rather, it served as an additional layer of information and data to enrich the study.

## Findings and discussion

### That’s what I signed up for

The first-order theme that emerged from the interviews was the early awareness of the magnitude of the fandom in their partners’ identities and daily lives. Because it is not visible to others, a sports fan’s devotion may be somewhat concealed. Not all interactions with friends or following the news about a favorite team or player occur publicly; instead, they frequently happen privately and according to the individual’s time preferences. Match viewing, on the other hand, necessitates continuous and lengthy time that is scheduled on a regular basis. However, it is done approximately once per week during the game season. Nonetheless, the interviewees noted that it was brought up by their partners early in the relationship and even during the first date. As Sarah mentioned:

Already on our first date, he presented it in such a way that it is an issue and that he will not give it up for anyone. I respect that. I try to respect that because I understand that it is important that everyone has their own things in a relationship. It is good that he was open about it from the beginning so I would know what I am getting into.

Because this issue was present from the beginning, it resulted in two important perceptions. First, because they decided to start the relationship after learning about their partner’s devotion, they saw it as an inherent part of who he is. Therefore, they do not necessarily see it as a personality flaw but rather yet another quality of their significant other. Helen even describes it as a virtue:

From the beginning, I knew that it is something that fills him and gives him confidence, and I think that confidence stays with him in other aspects of life, such as at work, being a good salesman. So I see it in a good way, having a hobby that gives so much and allows him to develop as well.

Maya articulated a similar viewpoint:

I think it points to some passionate component of his character. I can even say it was kind of a turn-on to see the intensity, his ability to show emotions, and his willingness to commit to something.

Second, this early awareness makes them feel like they are unable to criticize it. As Ruthy said:

We have known each other since high school. I am aware of this problem of his. Even back then, we used to argue about it. So, I knew what I signed up for, I guess I can not really complain.

Maya shared the same viewpoint:

Mistakes were made! (laughing). Even though I may not have understood the gravity of the situation right away, I was aware of its existence. Honestly, I thought it was something I could live with, and here I am doing just that. I can not get mad at him right now or accuse him of something that was there from the beginning. Not after 6 years.

According to the findings, even though this behavior significantly impacts the interviewees’ lives, they do not perceive it as a significant barrier to a successful partnership. They contend that they occasionally feel they must avoid casting this characteristic in a particularly unfavorable light because doing so would imply that they do not respect a significant aspect of their partner’s personality. The early recognition of a partner’s deep-seated fandom and subsequent adaptation align with contemporary psychological understanding. Cognitive dissonance theory suggests that individuals strive for internal consistency and adjust their cognitions when faced with dissonance-inducing information, such as a partner’s intense fandom, to reinstate cognitive equilibrium (Festinger, 1962; Harmon-Jones et al., 2015). This sheds light on why interviewees view their partners’ fandom as a fundamental and positive attribute. Additionally, the principle of selective perception emerges as interviewees frame their partners’ fandom in a positive light, emphasizing its contribution to personal growth or as a virtue, likely employing this biased perception as a coping strategy to sustain relationship harmony (Bruner, 1957; Hirt and Clarkson, 2011). The sunk cost effect may explain interviewees’ hesitation to critique their partners’ fandom, as their investment in the relationship despite knowing the extent of the fandom leads them to avoid expressing negative viewpoints that may question their initial decision to enter the relationship (Arkes and Blumer, 1985; Ronayne et al., 2021).

### Fandom as a gendered activity

In many Western societies, women participate less frequently in sports than men do (Deaner et al., 2016), and they are also less involved in the world of sports fandom (Pfister, 2010). This can be attributed to social norms that associate sports-related activities with masculinity (Hertzog and Lev, 2019). The participants in the current study are aware of this widely held perception of sports and can relate to it in the context of their partners’ fervent support. The interviewees occasionally describe certain behaviors as masculine while adopting a feminine stance toward others. For example, as Helen says:

I do not really care about sports. I just can not relate to that. I prefer cooking for my kids, playing with them, helping them with homework. Be a mom. I admit that when he watches his team, he is not exactly a dad, and because of this, I do not want to get into this whole thing. I do not want sports to take over the other things I do.

A similar attitude can be found in Mia’s words:

When he watches football, I do something of my own, like taking a long shower. I really love. I also take care of the house, like washing dishes, doing laundry, etc. I know how important it is to him, so I really try to let him do his thing, and I take care of the housework.

In addition to the apparent distinctions between each partner’s activities, the gendered nature of sports fandom often includes a component of commitment or exclusivity. For instance, the general perception is that watching sports is an activity that must be undertaken without interruption, often rendering the partner physically or mentally unavailable. In contrast, the wife can juggle family responsibilities, such as caring for children, while simultaneously engaging in other leisure activities. As Nily puts it:

Yesterday I made plans to meet my friend in a café. In the end, I found myself meeting with her at my place instead because he wanted to go and watch the game with a friend of his, and someone had to watch over the kids. And above all, it was not even Beitar’s (Jerusalem) game, it is not his team. It drove me crazy.

Under the gendered lens of fandom, the interviewees point to somewhat chauvinistic behavior when it comes to practicing fandom. Many of them reported that their partners actively discourage them from participating in their fandom activities, including watching games together. Ruthy comments on this:

He never gets me into the world of basketball or shares stuff related to that with me, so I just ignore it. He’s convinced that I will not understand or will not be interested, so he’s not really trying to involve me.

Throughout the interviews, there were numerous instances where interviewees brought up the perceived inability to comprehend the sport in-depth. For example, Nurit said:

I can sit and watch football with him, but he does not always go along with it, especially when he is with friends. At the last World Cup, all his friends came to our house, and I wanted to sit with them for a while. I said something about Messi, and my husband’s friend said that I was just interested in the looks of the players and not because I am interested in the World Cup. After that, I decided not to sit with my husband’s friends anymore because they look down on me.

Eden adds,

He is truly not a chauvinist, but he turns into one as soon as it is about sports. I do not know how come, but he believes that women can not understand basketball or football just like men can not understand shopping or cooking. He knows how I feel about these primitive views. We fight quite a bit about it.

In the case of Mia’s relationship, it does not even matter that she has been an active fan for many years:

I am a fan of Maccabi Tel Aviv Football Club. It was part of my childhood. It was something my father and I did together. It was fun to go with him to watch the games, even though I was the only girl. All of his friends came with their sons. But my husband, well, he thinks it’s a men’s only zone. He had several comments about it. It does not come naturally to him that I know stuff. Whenever I say something I am sure of, I feel like he’s testing me. Like he thinks he knows better. As if I should be given his approval to even talk about it.

Participants describe their partners as adopting somewhat different personas while practicing their fandom. There seems to be a clear transition from their typical behaviors in daily life to those exhibited while engaging in fandom, which also influences their attitudes toward their partners. It appears to be a setting in which their partners feel free to adopt certain attitudes and behaviors. One participant compared this to her partner’s out-of-character actions in the stands, where he shouts, curses, chants, and even cries—things he never does under ordinary circumstances. The experiences shared by the interviewees are consistent with extensive contemporary research (Esmonde et al., 2015; Lenneis and Pfister, 2015; Warner and Dixon, 2015) that highlights societal norms associating sports with masculinity. This bias results in limited participation of women in sports and their exclusion from sports fandom (Allison and Knoester, 2021). The accounts provided by the interviewees shed light on the chauvinistic behavior exhibited by their partners, who discourage their involvement in sports fandom activities, reflecting prevailing gender role expectations (Grappendorf et al., 2023). Moreover, the findings allow us to discern how women’s knowledge and interest in sports are often undermined or dismissed, perpetuating biased perceptions within sports fandom (Toffoletti, 2017; Grappendorf et al., 2023). These findings underscore the existence of gender-based disparities and prejudices in sports fandom and emphasize the need to address the underlying inequalities.

### The good, the bad, and the ugly

The third-order theme that emerged from the analysis concerns the realistic perspective of relationship dynamics. The interviewees describe their partners’ fandom as a devotion that occupies a substantial portion of their lives. Simultaneously, they view it as a hobby that empowers their partners and can occasionally become a shared experience. In this regard, the women interviewed for this study note that fandom, and especially watching games, can present an opportunity for joint marital activity. Helen describes her efforts in this matter:

I stay in the living room and watch it (the game) with him. I want to be with him after spending the whole day separated. And I keep asking questions and show interest in the game. I learn slowly! I will not say that I fully understand but I am getting better. Sometimes he really wants me to sit next to him and watch it together as if he wants it to be our hobby.

Similarly, Dinah describes how watching sports can be turned into a social gathering:

There are games, for example, the Football World Cup. It was like a holiday here! We went to the grocery store together, bought snacks, and his friends came to our place to watch the games. We brought a projector, speakers. do not ask. I also watched with them, and some other wives too. It was a festival. I really enjoyed that period, I felt that there was something that connects us, something we are waiting for together.

On the other hand, the interviewees understood that their partners’ primary motivation for shared activities was their interest in sports. Therefore, they knew their partners would pursue their fandom regardless. This understanding led to a conflict: should they embrace their partners’ hobby, or let them engage freely in it, even if it comes at the expense of the relationship? As the findings suggest, the wives often accept that their husbands will be preoccupied with their fandom. Mia provides some illustrative examples:

He helps in the house chores, but when there’s a match on, it is his priority, without a doubt. On the other hand, he knows that he must help in any way possible right after, and he knows he can not avoid it. So I put aside things I want him to do, and he does.

And Ruthy adds:

There’s an unwritten agreement between us. He does not always help with the kids because he watches basketball, so when I have Pilates, he has to take care of the kids. We respect each other’s hobbies and activities. The catch is that his’ takes over much more.

Participants’ attitudes are ambiguous as a result of this acceptance. They describe a wide variety of feelings, from apathy and avoidance to frustration. Yael stated this:

It is frustrating, but I got used to it… It is not the most fun or comfortable thing in the world to be married to someone who is an ardent sports fan that invests a lot of energy and time in it. It is hard, and you have to learn to live with it.

Alongside the opportunity to spend time together, or understanding that there will be times of separation, some interviewees claimed that their partner’s fandom could be detrimental to the relationship. In other words, engaging in fandom does not only come at the expense of the relationship, but it might also hinder mutual support or strain their financial situation. Sarah provides an example of this:

He loves his club. So, if the team wins, he is feeling high, if it loses, he’s down. like a depression. And let’s say, once a year he flies abroad with his friends, and deep inside, in my heart, I go crazy. He travels to these “sport-things” and puts me in a lower priority because it means less money for us to fly and travel.

It should be stressed that this circumstance often arises when the interviewees believe their partner’s interest has escalated into an obsession or addiction, a topic explored in the following theme. The insights shared by the partners offer an invaluable perspective into the complex dynamics of relationships influenced by intense fandom which often result in what Lev and Zach (2018) referred to as “conditional support.” While their willingness to participate in shared activities and support their significant other’s passion is evident, they face daunting challenges when fandom becomes dominant. Previous research, including studies by Wakefield and Wann (2006) and Wann and Branscombe (2010), has highlighted these dynamics, pointing to conflicts, frustration, and feelings of neglect when a partner’s fandom takes precedence. These challenges echo in the experiences shared by interviewees, which are further substantiated by research from Lim and Putnam (2010). They, too, found partners of avid sports fans wrestling with similar issues, with fandom frequently overshadowing the relationship. Further, this complex interplay underscores the necessity of striking a balance between personal passions and shared responsibilities, which is crucial for sustaining a healthy relationship. Central to achieving this balance is the pivotal role of open communication, mutual understanding, and compromise, as these factors are integral to fostering a harmonious partnership amidst intense fandom (Luellen and Wann, 2010; Shtudiner et al., 2022).

### Sport fandom as an addiction

It should be mentioned that the analogy of intense fandom being an addiction arose spontaneously during the interviews. Usually, this term was intended to highlight the magnitude of the emotional, financial, and temporal resources invested in the fandom. Helen pointed out the difference between “normal” and “addicted” fan:

He is definitely an addict. For example, I am also a fan but it is not the same thing at all. I have boundaries, and I take things in proportion. He is not. Nothing else is important if his club is playing, only the game. It is not a normative behavior for a married 42 years old with three kids. This addiction regularly comes at my expense.

Sarah adds to that:

I know it is an exaggerated comparison, but it is like it is some drug he needs to use to get through the week. So, he will not suffer from withdrawals. If he does not go to the games, the whole week is ruined. I think the addiction is also reflected in the fact that he is constantly checking the online sports sites, it is always there.

The interviewees described addiction in two ways: a sudden change in mood and its implications for oneself and others. The first is the impact of the games’ outcomes on mood and behavior. Ruthy stated:

He does not get enough sleep because of it, and then he’s not concentrated on work. Sometimes he’s having trouble falling asleep after a bad result.

And Nily added:

If the team loses it is as if a disaster has happened. Like someone’s died. The kids and I just know not to talk to him until the next day. He goes to bed nervous; I have not seen anything like that. Like an addiction, it affects him that much.

According to the findings, changes in mood among fans are directly and immediately influenced by game outcomes. This predictable pattern of emotional fluctuations makes it possible for individuals to manage their emotional responses. Another aspect of addiction is its impact on those around them. Mia elaborated on how her partner’s fandom affects her and her schedule:

This addiction comes at my expense in a lot of cases, if there’s something on TV related to his club, a match, a press conference, I do not know what, anything, then my stuff are automatically dismissed by him, that is, if I have an appointment or I want or need to do something, I have to give it up and be with the kids. because his club and football are always first priority. We had a lot of quarrels because of that.

Being the partner of an individual exhibiting addictive tendencies toward fandom, as evident from our interviews, can be a significant emotional challenge, much like living with a substance addict (Orford et al., 2010). The findings can demonstrate how living with a partner who is addicted to fandom can be emotionally challenging, like living with a substance addict. Partners often face the fan’s compulsive dedication to the fandom, leading to neglect of family responsibilities and strained relationships due to the disproportionate allocation of resources (Fuschillo, 2020). Furthermore, the fan’s withdrawal-like symptoms can disrupt domestic harmony and personal wellbeing, creating an environment of instability (Rudski et al., 2009). The emotional turbulence resulting from a team’s loss can mirror the psychological distress associated with addiction, further impacting the relationship dynamics (Friedman, 2020). When fans also engage in risky behaviors such as gambling and substance abuse, it not only harms the fan but also cultivates an unpredictable, potentially unsafe environment for their partners (Wann and James, 2018; Archer, 2021). Despite these challenges, it should be pointed out that not every passionate fan demonstrates addictive patterns. However, when fandom significantly disrupts daily life and interpersonal relationships, seeking professional mental health support can be beneficial for all involved parties (Wakefield and Wann, 2006). After all, compulsive fandom dedication can lead to an unequal distribution of responsibilities, neglect of familial duties, and strained relationships due to an imbalanced commitment of resources (Orford et al., 2010). This addiction-like behavior often results in fans neglecting their partners and children, as well as spending excessive amounts of money on fandom-related activities (Fuschillo, 2020). Furthermore, fans may experience withdrawal-like symptoms when deprived of their fandom, leading to disruptions in domestic harmony and negative effects on personal wellbeing (Rudski et al., 2009). These symptoms can include irritability, anxiety, and depression (Weinstein et al., 2017). Additionally, as demonstrated, the emotional upheaval that fans experience following their team’s loss can further strain interpersonal dynamics, causing feelings of sadness, anger, and frustration (Friedman, 2020).

## Concluding remarks

The primary objective of this study was to gain a comprehensive understanding of the perspectives and emotions expressed by the spouses and girlfriends of avid sports fans in relation to their enduring relationships. We intended to highlight the possible outcomes that emerge when individuals become deeply engrossed in a competitive sport, showcasing intense emotional bonds, wide-ranging knowledge, steadfast loyalty, and active engagement in fan-related activities.

As the findings indicate, the concept of social identification, as introduced by Tajfel and Turner (1978), is central to sports fandom, underscoring the psychological link between fans and their preferred team or sport. The fans in this study demonstrated high levels of identification revealing a deep sense of loyalty, knowledge, and attachment to their teams. This identification with a particular team or sport becomes a crucial part of a fan’s identity, promoting a sense of belonging and fostering a community within the larger fan base. This association with a specific team or sport becomes a crucial part of a fan’s identity. As a result, the identification process with a sports team holds emotional significance, with fans’ self-esteem often tied to this affiliation. In this context, the inflexible nature of identification not only risks jeopardizing romantic relationships but also mirrors a disconcerting reality that perpetuates gender inequality while marginalizing and undermining women’s agency.

Furthermore, it is important to acknowledge that these findings are deeply embedded within a specific cultural context. The Israeli army, as a formidable institution that shapes gender disparities and power imbalances, perpetuates male dominance both internally and externally. Given that militarism occupies a foundational role in Israeli identity (Sasson-Levy and Hartal, 2018), the entrenched machismo culture within the military sustains and propagates the notion of male “superiority” and female dependency. This dynamic is also highly pertinent within recreational sports (Lev and Hertzog, 2022), where the biological and physiological elements of such activities contribute to their perception as inherently “natural” (Hertzog and Lev, 2019).

The social critique voiced by the interviewees against non-traditional gender behavior exhibited by their partners serves as a warning, aiming to protect and maintain male dominance in sports, hence reinforcing their unquestioned supremacy. Consequently, latent aggressive behavior in men is perceived as a means to preserve their exclusive domain over sports fields. It is vital to acknowledge that while such behaviors have been linked to relationship difficulties among fans, not all ardent fans display aggressive or addictive behaviors. However, if the intensity of fandom significantly interferes with daily life and relationships, it is advisable for all parties to consider seeking professional mental health support (Wakefield and Wann, 2006).

The findings presented in this study contribute to the psycho-sociological field of sports fandom, especially in exploring the connection between attitudes and emotions expressed by fans’ partners in relation to their enduring relationships. As such, this study’s implications should foster a deeper understanding and heightened awareness among avid sports fans prior to committing to serious relationships or formalizing existing ones. Romantic relationships in which one partner exhibits fervor necessitate a critical reassessment and reflection. Extending the discourse about striking a balance between passion and relationship responsibilities, relationship psychology offers a powerful tool for managing these challenges: the concept of a shared meaning system. As suggested by Gottman et al. (2015), this involves constructing a collective narrative that accommodates the needs and interests of both partners, thereby forging an interconnected network of shared goals and values.

In closing, it is important to note that the present study was conducted with a sample size of twelve fan spouses and girlfriends. Given that the expression of sports fandom within romantic relationships can differ across geographic and socio-economic contexts, the generalizability of this study is limited. Further research should encompass diverse backgrounds, examining various geographic locations and social milieus (e.g., religious groups and rural regions). Additionally, conducting a study specifically focusing on female sports fans in relationships with non-avid fans could provide an interesting counterpoint. Despite these limitations, we believe that the findings reported herein offer valuable insights into understanding the complex nature of fans in romantic relationships as a universal phenomenon.

## Data availability statement

The raw data supporting the conclusions of this article will be made available by the authors, without undue reservation.

## Ethics statement

The studies involving humans were approved by the Ethics Committee of Ohalo College, Israel (04/2019, code 01221). The studies were conducted in accordance with the local legislation and institutional requirements. The participants provided their written informed consent to participate in this study.

## Author contributions

All authors contributed equally to all stages of the manuscript, contributions encompass the conception and design of the research, data acquisition and analysis, interpretation of results, drafting and critical revision of the manuscript, and final approval for submission.
